# The prevalence and prognosis of next‐generation therapeutic targets in metastatic castration‐resistant prostate cancer

**DOI:** 10.1002/1878-0261.13320

**Published:** 2022-10-20

**Authors:** Jian Pan, Jinou Zhao, Xudong Ni, Hualei Gan, Yu Wei, Junlong Wu, Tingwei Zhang, Qifeng Wang, Stephen J. Freedland, Beihe Wang, Shaoli Song, Dingwei Ye, Chang Liu, Yao Zhu

**Affiliations:** ^1^ Department of Urology Fudan University Shanghai Cancer Center China; ^2^ Shanghai Genitourinary Cancer Institute China; ^3^ Department of Oncology, Shanghai Medical College Fudan University Shanghai China; ^4^ Department of Nuclear Medicine Fudan University Shanghai Cancer Center China; ^5^ Department of Surgery, Division of Urology and Samuel Oschin Comprehensive Cancer Institute Cedars‐Sinai Medical Center Los Angeles CA USA; ^6^ Urology Section, Department of Surgery Veterans Affairs Medical Center Durham NC USA; ^7^ Department of Pathology Fudan University Shanghai Cancer Center China

**Keywords:** abiraterone, HRR, metastatic castration‐resistant prostate cancer, prognosis, PSMA, PTEN

## Abstract

The success of the PROfound, IPATential150, and TheraP trials promoted the transition from sequential treatment to therapeutic targets (TTs)‐guided precision treatment in metastatic castration‐resistant prostate cancer (mCRPC). The objective of this study was to evaluate the prevalence and prognostic value of TTs from these three trials. All included Chinese mCRPC patients underwent circulating tumor DNA (ctDNA) sequencing, PTEN status assessment, and dual‐tracer [^68^Ga‐prostate‐specific membrane antigen (PSMA) and ^18^F‐fluorodexyglucose (FDG)] positron emission tomography/computed tomography (PET/CT). Previous treatment with cabazitaxel, Lu‐PSMA or olaparib was unallowed. Patients with known significant sarcomatoid or spindle cell or neuroendocrine small cell components were also excluded. TTs were defined as positive as follows: (a) high PSMA and no PSMA−/FDG+ disease on dual‐tracer PET/CT scans; (b) defects in homologous recombination repair (HRR) genes in ctDNA; and (c) loss of PTEN immunohistochemistry staining in tumor tissue. The prevalence and prognostic value on progression‐free survival (PFS) of TTs were evaluated. A total of 106 consecutive mCRPC patients were included. The prevalence of positive PET/CT, HRR defect, and PTEN loss was 30%, 29% and 16%, respectively. Sixty‐three patients had at least one TT. Metastatic volume (odds ratio = 5.0; *P* = 0.017) was the only independent factor of positive TT in multivariate analysis. Seventy‐four patients received abiraterone after TT screening. Patients with positive PET/CT (*P* = 0.011) and HRR defect (*P* = 0.002) had a significantly shorter PFS after receiving abiraterone than patients with negative TTs. However, PTEN status was unrelated to PFS, which may be due to a less number of patients with PTEN loss (*P* = 0.952). Overall, patients with any positive TTs had a significantly shorter PFS after abiraterone than patients with negative TTs (*P* = 0.009). Nearly 60% of Chinese patients with mCRPC who had a poor prognosis on abiraterone were candidates for precision treatments based on the specific criteria of TTs.

## Graphical abstract

Results of the PROfound, IPATential150 and TheraP trials were evaluated to determine the frequency of mCRPC patients who had therapeutic targets. All included mCRPC patients underwent ctDNA sequencing, PTEN status assessment and dual‐tracer (^68^Ga‐PSMA and ^18^F‐FDG) PET/CT. Nearly 60% of patients had positive therapeutic targets. In addition, patients with positive therapeutic targets had a shorter progression‐free survival under abiraterone treatment.

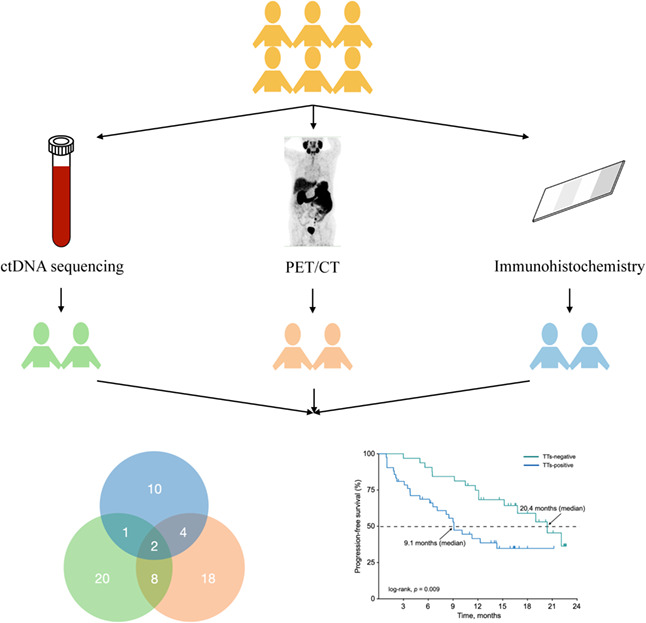

**Abbreviations**
ADT: androgen deprivation therapy
cfDNA: cell‐free DNA
CI: confidence interval
ctDNA: circulating tumor DNA
ECOG: Eastern Cooperative Oncology Group
FDG: fluorodexyglucose
HR: hazard ratio
HRR: homologous recombination repair
ICD: international classification of diseases
IHC: immunohistochemistry
IQR: interquartile range
mCRPC: metastatic castration‐resistant prostate cancer
OS: overall survival
PARPi: poly ADP‐ribose polymerase inhibitor
PCa: prostate cancer
PET/CT: positron emission tomography/computed tomography
PFS: progression‐free survival
PSA: prostate‐specific antigen
PSAdt: PSA doubling time
PSMA: prostate‐specific membrane antigen
RECIST: response evaluation criteria in solid tumors
SNP: single nucleotide polymorphism
TT: therapeutic target

## 1 Introduction

Prostate cancer (PCa) is a major public health problem among men, accounting for 14% of male malignancies [1]. Even though metastatic PCa responds to androgen deprivation therapy initially, in time, most patients will eventually progress and become castration‐resistant [2]. Docetaxel, cabazitaxel, sipuleucel‐T, abiraterone, enzalutamide, and radium‐223 are recommended for metastatic castration‐resistant prostate cancer (mCRPC) in National Comprehensive Cancer Network (NCCN) PCa guidelines, but the application of these therapies is rarely based on therapeutic targets (TTs) [3].

The management of mCRPC is undergoing a rapid transformation from sequential treatment of overall patients to selective treatment of those with specific targets. First, in Phase III PROfound trial of mCRPC patients with homologous recombination repair (HRR) alterations who had failed prior AR targeted therapy (abiraterone or enzalutamide), median overall survival (OS) was 17.3 vs. 14.0 months [hazard ratio (HR), 0.79; 95% confidence interval (CI), 0.61–1.03] with olaparib and the other AR targeted agent, respectively [4]. The TheraP trial used the ^68^Ga‐PSMA and ^18^F‐FDG (dual‐tracer) PET/CT to enroll patients with high PSMA uptake and no PSMA−/FDG+ disease. They found a significantly higher prostate‐specific antigen (PSA) response (66% vs. 37%, *P* < 0.001) of Lutetium‐177 [^177^Lu]Lu‐PSMA‐617 therapy [5]. The IPATential150 trial showed that ipatasertib and abiraterone significantly prolonged radiographic progression‐free survival (PFS) in mCRPC with PTEN loss in immunohistochemistry (IHC) staining (HR, 0.65; 95% CI, 0.45–0.95), but failed to benefit those with PTEN intact tumor [6]. The promising efficacy of these TTs confirmed the hypothesis that using next‐generation sequencing, specific imaging criteria or IHC could enrich a subset of patients who are mostly likely to benefit from targeted therapy. However, several important questions should be answered before establishment an individualized treatment approach. First, can we develop an integrated TT screening platform to identify patients eligible for specific treatments, and how many patients could benefit from this platform? Second, whether TTs provide prognostic value in circumstances where the best evidence is still the standard treatment? The information is strongly needed not only for treatment recommendation, but also for future clinical trial design.

To evaluate the frequency of mCRPC patients who would have qualified for these precision trials, we conducted this study to: (a) estimate the prevalence of TTs from PROfound trial, TheraP trial and IPATential150 trial in Chinese mCRPC patients; (b) to evaluate the prognostic value of TTs.

## 2 Materials and methods

### 2.1 Study design and participants

We enrolled 141 consecutive men with mCRPC treated at Fudan University Shanghai Cancer Center (FUSCC) between April 2019 and March 2021. Pathological review was performed to confirm the diagnosis of prostate adenocarcinoma. Patients with secondary malignancy history within 3 years or infectious disease within 90 days were excluded. Additional exclusion criteria were as follows: prior treatment with cabazitaxel, Lu‐PSMA, or olaparib; prostate cancer with known significant sarcomatoid or spindle cell or neuroendocrine small cell components. The definitions of sarcomatoid and small cell carcinoma components were both based on the international classification of diseases for oncology approved by the International Agency for Research on Cancer/World health Organization committee. The sarcomatoid component [international classification of diseases (ICD)‐O code: 8572/3] often consisted of a non‐specific malignant spindle‐cell proliferation. Among the specific mesenchymal elements were angiosarcoma, rhabdomyosarcoma, leiomyosarcoma, chondrosarcoma, liposarcoma, osteosarcoma, or multiple types of heterologous differentiation [7, 8]. The epithelial component showed immunoreactivity for prostatic acid phosphatase, PSA, cytokeratin, and whereas the soft tissue component showed immunoreactivity for various mesenchymal markers. On IHC, the small cell component (ICD‐O code: 8041/3) was positive for one or more neuroendocrine markers (e.g. CD56, chromogranin, and synaptophysin) in almost 90% of cases. In some cases, positivity was noted for high‐molecular weight cytokeratin and p63, which a typically negative in prostatic carcinoma [9, 10]. Dual‐tracer PET/CT and blood tests were performed within 1 month after enrolment. The demographic and clinical information of each participant was recorded. This study was performed after the approval of the Human Ethics Committee of FUSCC and was conducted following the Declaration of Helsinki. Written informed consent was obtained from all patients.

### 2.2 Sample processing, DNA sequencing and bioinformatics

The whole blood samples collected in the Streck cell‐free DNA (cfDNA)‐free blood collection tubes were centrifuged at 16 000 ***g*** for 10 min at 4°C to separate the buffy coat and plasma. Then, according to the manufacturer's protocol, cfDNA was isolated from the plasma using the QIAamp Circulating Nucleic Acid Kit (Qiagen, Hilden, Germany). At the same time, germline DNA from white blood cells was isolated using DNeasy Blood and Tissue Kit (Qiagen, 69504). Detailed methods were performed in Burning Rock Biotech, a College of American Pathologist‐accredited/Clinical Laboratory Improvement Amendments‐certified commercial clinical laboratory. We required 50 ng of cfDNA for next‐generation sequencing library construction. The indexed samples were sequenced on Nextseq500 (Illumina, Inc., San Diego, CA, USA) with paired‐end reads and average target sequencing depth of 10 000×. To identify the somatic variants, germline variants and the ctDNA fraction, we used methods published previously [11, 12]. Sequence data were mapped to the reference human genome by burrows‐wheeler aligner version 0.7.10 [13]. Local alignment optimization, duplication marking and variant calling were performed using genome analysis tool kit version 3.2 [14], and varscan version 2.4.3 [15]. Loci with depth less than 100 were filtered out using the varscan fpfilter pipeline. Base calling in plasma samples required at least eight supporting reads for single nucleotide variations and five supporting reads for insertion–deletion variations, respectively. Variants with population frequency over 0.1% in the ExAC, 1000 Genomes, single nucleotide polymorphism (SNP) database or ESP6500SI‐V2 databases were grouped as SNPs and excluded from further analysis. Remaining variants were annotated with annovar [16] and snpeff version 3.6 [17]. We analyzed DNA translocation by factera version 1.4.3 [18]. We analyzed targeted sequencing data from plasma cfDNA and matched leukocyte DNA samples by using a targeted panel to capture the exons of 15 genes associated with HRR: *ATM*, *BRCA1*, *BRCA2*, *BRIP1*, *CHEK1*, *CHEK2*, *BARD1*, *FANCL*, *CDK12*, *RAD54L*, *PALB2*, *PPP2R2A*, *RAD51B*, *RAD51C* and *RAD51D* [4]. Patients with one or more deleterious somatic or germline mutations in HRR genes were defined as HRRmt group. The rest were defined as the HRRwt group.

### 2.3 PET/CT imaging and image analysis

^68^GaCl3, eluted from ^68^Ge‐^68^Ga generator (IGG100, Eckert & Ziegler, Berlin, Germany) by slow injection of 5 mL of 0.1 m HCl, was added into the vial which contained 40 μg PSMA11 (Jiangsu Huayi Technology Co., Ltd, Changshu, China) and 0.5 mL NaAc, and the mixture was heated at 100°C for 10 min. Finally, the solution passed through a 0.22 μm pore size filter into the product vial. ^18^F‐FDG was produced automatically by cyclotron (Siemens CTI RDS Eclips ST, Knoxville, TN, USA) using Explora FDG4 module in our center [19]. The two radiotracers were not scheduled on the same day, and the interval was less than 5 days [20, 21]. ^18^F‐FDG PET/CT patients fasted for at least 6 h. Blood glucose levels before the injection of the tracer were lower than 10 mmol·L^−1^. The routine scan was initiated 60 min after administration of the tracer (3.7 MBq·kg^−1^). ^68^Ga‐PSMA PET/CT did not require fasting. ^68^Ga‐PSMA patients were asked to oral intake of 500 mL of water during a 2‐h period prior to acquisition (2.0 MBq·kg^−1^) without dietary preparation. A 60‐min interval was also adopted for uptake time [22]. PET/CT scans were performed using a Siemens mCT Flow PET/CT scanner (Siemens Healthcare, Knoxville, TN, USA). A non‐contrast‐enhanced CT scan was performed using the following parameters: slice thickness of 3 mm, increment of 2 mm, soft tissue reconstruction kernel, 120 keV. Immediately after CT scanning, a whole‐body PET (from the level of the skull base to the knee) was acquired in 3‐D (matrix 200 × 200). A multimodality computer platform (Syngo; Siemens Healthcare) was used for image review and manipulation. Transaxial, coronal, and sagittal reconstructions of CT, PET, and fusion PET/CT data for interpretation can be produced by this system. All PET/CT scans were reviewed and interpreted by three experienced nuclear‐medicine specialists who were blinded to result of histopathology/biopsy and follow‐up imaging. The reviewers reached a consensus in cases of discrepancy. Visual interpretation: Each reviewer will mark regions of suspected disease based on a two‐point scale: 0 as negative or 1 as positive. Only when a visually positive lesion is found in the region, it is graded as 1. Positive lymph nodes: only if the ^68^Ga‐PSMA or ^18^F‐FDG uptake is locally accumulated and higher than blood pool (mediastinal blood pool). Lymph nodes will be categorized as retroperitoneal, pelvic and distant depend on its location. Positive visceral lesions: only if the ^68^Ga‐PSMA or ^18^F‐FDG uptake is locally accumulated and higher than the background activity of surrounding involved organ or region. Positive bone lesions: only if the ^68^Ga‐PSMA or ^18^F‐FDG uptake is locally accumulated and higher than physiologic bone marrow. Bone lesion will be categorized as axial, limbs and pelvis skeleton depend on its location. Patients were defined as the PET/CT‐high group according to the imaging inclusion criteria of the TheraP trial [23]: with minimum uptake of SUV_max_ 20 at a site of disease, and SUV_max_ > 10 at sites of measurable disease ≥ 10 mm, and no PSMA−/FDG+ lesion. The rest were defined as the PET/CT‐low group. Patients were followed with conventional imaging every 3 months. Only imaging obtained at baseline was used to assess PSMA marker presence.

### 2.4 PTEN immunohistochemistry

Formalin‐fixed paraffin‐embedded tumor tissue was retrieved for IHC analysis. IHC was performed using the SP218 (1 : 200) PTEN antibody according to previous reports. PTEN status classification was based on the tumor with the lowest PTEN staining [24, 25]. Lesions were defined as PTEN loss status if they either showed a complete absence of PTEN staining or weak intensity staining compared with surrounding benign tissue or stroma in more than 50% of malignant cells with no specific cytoplasmic staining [26]. Patients with PTEN‐loss tumors were defined as the PTEN‐loss group, and the rest were defined as the PTEN‐intact group. The IHC results were independently analyzed by two experienced urologic pathologists blinded to clinical outcome data. A consensus would be reached when discrepancies occurred.

### 2.5 Statistical analysis

The HRRmt group, PET/CT‐high group, or PTEN‐loss group were also defined as TTs‐positive group. The rest were defined as the TTs‐negative group. The primary endpoint was to estimate the prevalence of the HRRmt, PET/CT‐high, and PTEN‐loss in Chinese mCRPC patients. The secondary endpoint was to evaluate the association between the status of TTs and the outcomes of treatment after enrollment. Treatment outcome was accessed using PFS. PSA testing was repeated every 4 weeks, and imaging evaluation was repeated every 12 weeks. PFS was defined as freedom from any of the following: death from any cause, the first evidence of PSA progression identified by an increase of at least 25% and > 2 ng·mL^−1^ after 12 weeks [according to Prostate Cancer Clinical Trials Working Group 3 (PCWG3)] [27], or radiographic progression using locally reported CT and bone scanning (Response Evaluation Criteria In Solid Tumors [RECIST] 1.1 and PCWG3 criteria for bone lesions). The Kaplan–Meier method was used to estimate PFS outcomes. The disease burden was classified into low volume and high volume according to the CHAARTED trial [28]. PSA doubling time (PSAdt) was calculated using the three consecutive rising PSA levels before enrolment. Categorical data are shown as frequencies and percentages and continuous data are shown as medians and interquartile ranges (IQR). The chi‐square test was applied to analyze the difference in categorical data. Logistic regression was performed to estimate the association of prevalence with clinicopathological features (such as Gleason score, metastases volume, stage at diagnosis, etc.).

In the multivariate analysis, patients were categorized into three risk groups based on the number of the following six risk factors [LDH, Alb, ALP, metastasis status of liver, ECOG performance status, time from start of initial androgen deprivation therapy (ADT) to initiation of abiraterone] that were assessed for significant association with OS in mCRPC patients treated with abiraterone from a well‐established prognostic prediction model [29]: patients with 0 to 1 risk factor were in the good prognosis category; patients with 2 to 3 risk factors were in the intermediate prognosis category; patients with at least 4 risk factors were in the poor prognosis category. HR was generated by cox regression analysis and adjusted for history of docetaxel, prognosis category and status of TTs. A type I error of 0.05 (two‐sided) was used to define statistical significance. Visualization and statistical analysis were performed using r 3.6.1 platform.

## 3 Results

### 3.1 Baseline demographics

**Fig. 1:**
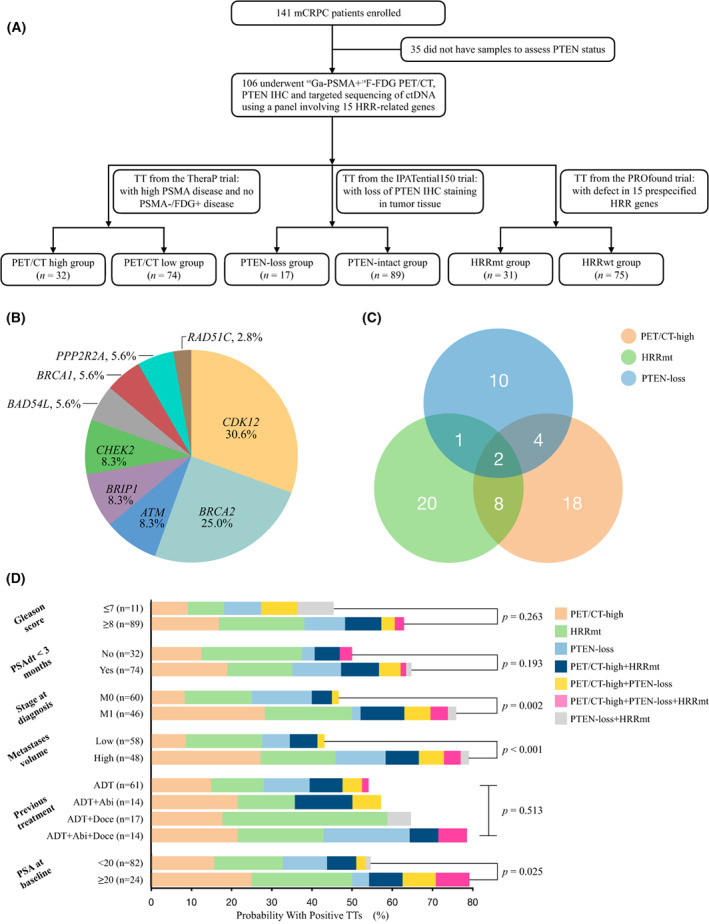
The prevalence and overlap of three TTs in our cohort. (A) Flowchart of this study. (B) Distribution of genomic alterations in HRR genes. (C) the Venn diagram represents the overlap of TTs. (D) the prevalence of TTs according to various clinicopathological features. Fisher's exact test was performed to assess statistical differences between the subgroups with different clinicopathological features. ctDNA, circulating tumor DNA; HRR, homologous recombination repair; IHC, immunohistochemistry; mCRPC, metastatic castration‐resistant prostate cancer; TTs, therapeutic targets.

Of 141 Chinese mCRPC participants, 35 were excluded from analysis because of eligibility criteria of TT‐assessment was not fulfilled (Fig. 1A). The median age was 68 years (IQR 63–73), and 89 (89%) patients with Gleason score ≥ 8 (the Gleason score was not applicable for six patients who underwent tumor biopsy after ADT). The median PSA level before enrolment was 5.2 ng·mL^−1^. Seventy‐four (70%) patients had a PSAdt of less than 3 months (Table 1).

**Table 1:** Baseline demographics. ECOG, Eastern Cooperative Oncology Group; IQR, interquartile range; mCRPC, metastatic castration‐resistant prostate cancer; PSA, prostate‐specific antigen; PSAdt, PSA doubling time.

Characteristic	Patient number (*n* = 106)
Median age at mCRPC, years (IQR)	68 (63–73)
Median PSA at baseline, ng·mL^−1^ (IQR)	5.2 (3.3–14.3)
Gleason score^a^, *n* (%)	
≤ 7	11 (11)
≥ 8	89 (89)
ECOG performance status, *n* (%)	
0	61 (58)
1	43 (40)
2	2 (1)
PSAdt < 3 months, *n* (%)	74 (70)
Site of metastatic disease, *n* (%)	
Visceral: lung or liver	6 (6)
Bone	81 (76)
Non‐reginal lymph node	54 (51)
Metastases volume, *n* (%)	
High	48 (45)
Low	58 (55)
Previous treatment, *n* (%)	
ADT	61 (58)
ADT + docetaxel	17 (16)
ADT + abiraterone	14 (13)
ADT + abiraterone + docetaxel	14 (13)

a The Gleason score is not applicable in 6 patients who had tumor biopsy after ADT.

### 3.2 The prevalence of TTs

The enrolment of patients participating in our study is depicted in the flowchart (Fig. 1A). HRR mutations were detected in 29% (31/106) of patients. The most frequently altered genes were *CDK12* (*n* = 11, 10%), *BRCA2* (*n* = 9, 8%), *CHEK2* (*n* = 3, 3%), *BRIP1* (*n* = 3, 3%) and *ATM* (*n* = 3, 3%; Fig. 1B). Based upon PROfound trial [4], 15 (14%) patients would have qualified based upon HRR mutations alone for cohort A (with aberrations in *ATM*, *BRCA1*, and *BRCA2*) and 16 (15%) patients would have qualified for cohort B (with aberrations in 12 other HRR genes). PTEN‐loss status was observed in 16% (17/106) of the FUSCC cohort. According to the specific PSA criteria of the TheraP trial (PSA ≥ 20 ng·mL^−1^), we divided the FUSCC cohort patients into two groups. Overall, 50% and 24% of the patients met the TTs criteria of the TheraP trial in the PSA ≥ 20 and < 20 ng·mL^−1^ groups, respectively (Table S1). A total of 66% (70/106) and 23% (24/106) of patients were excluded because of the low expression at all sites and the PSMA−/FDG+ disease, respectively. In addition, 19% (20/106) of patients had both low expression at all sites and PSMA−/FDG+ disease.

Fifty‐nine percent (63/106) of patients had at least one positive TT, and 14% (15/106) of patients had two 2 or more positive TTs (Fig. 1C). The prevalence of positive TTs was pairwise independent (*P* > 0.50; Table S2). Univariate analysis demonstrated a statistically positive correlation between higher stage at initial diagnosis (*P* = 0.002), higher metastatic volume (*P* < 0.001), and higher PSA (*P* = 0.025) and the greater likelihood of a positive TT. Multivariate analysis identified metastatic volume (odds ratio = 5.0; 95% CI, 2.1–12.0; *P* = 0.017) as the only independent factor associated with a higher a probability of a positive TT (Fig. 1D).

### 3.3 Exploratory progression‐free survival analyses

**Fig. 2:**
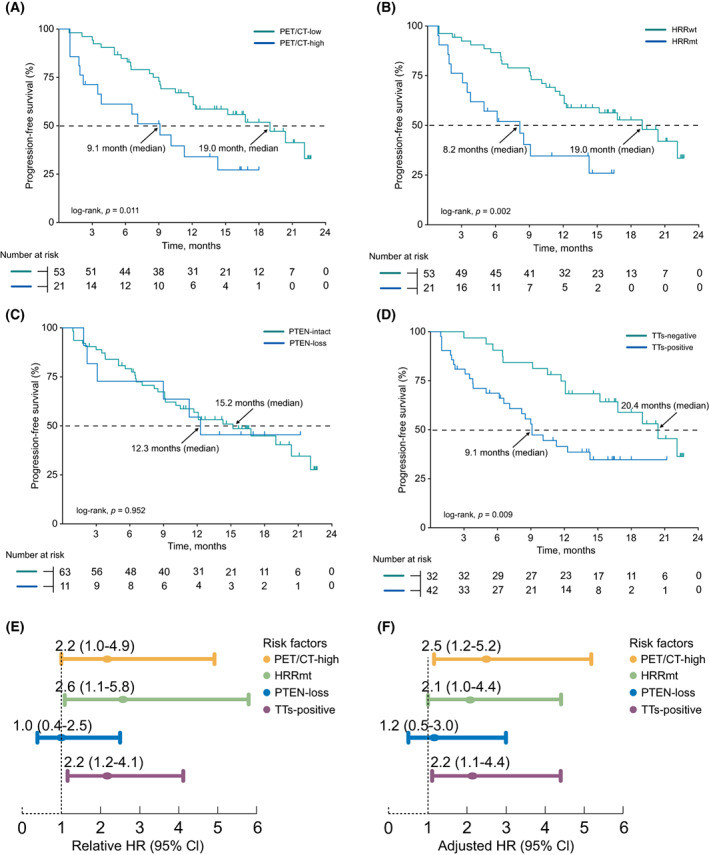
Progression‐free survival on abiraterone treatment and hazard ratios of TTs (*n* = 74). Kaplan–Meier survival curves according to the status of ‘PET/CT' (a), ‘HRR' (B), ‘PTEN' (C), and ‘TTs' (D). Univariate (E) and adjusted (F) HRs of PET/CT‐high, HRRmt, PTEN‐loss, and TTs. CI, confidence interval; HRR, homologous recombination repair; HRs, hazard ratios; TTs, therapeutic targets.

Seventy‐four patients received abiraterone plus prednisone after enrolment. The number of patients receiving other treatments (^177^Lu‐PSMA‐617 [1%, 1/106], pembrolizumab [1%, 1/106], olaparib plus abiraterone [1%, 1/106], radiotherapy [3%, 3/106], docetaxel plus carboplatin [4%, 4/106], olaparib [10%, 10/106], docetaxel [10%, 11/106]) was relatively small which unallowed the PFS analysis (Table S3). Therefore, we just performed an exploratory analysis to evaluate the prognosis value of TTs in patients using abiraterone as the following treatment after enrolment. At the last follow‐up, 54% (40/74) of patients had progressed during treatment with abiraterone. The median follow‐up was 18.0 (15.7–21.0) months. A significant association was seen in the PET/CT‐high group, which had worse PFS (median 9.1 vs. 19.0 months; *P* = 0.011; Fig. 2A). Similarly, those men with HRR mutations had significantly worse PFS (median 8.2 vs. 19.0 months; *P* = 0.002; Fig. 2B). However, PTEN loss status was unrelated to PFS, which may be due to a low number of patients with PTEN loss status (median 12.3 vs. 15.2 months; *P* = 0.952; Fig. 2C). Importantly, we observed a significant association between PFS and the presence of any of the three TTs (median 9.1 vs. 20.4 months; *P* = 0.009; Fig. 2D).

In the univariate analysis, the HR of TTs for PFS suggested that the PET/CT‐high (HR, 2.2; 95% CI, 1.0–4.9), HRRmt (HR, 2.6; 95% CI 1.1–5.8) and TTs‐positive (HR, 2.2; 95% CI, 1.2–4.1) groups had a higher risk of progression (Fig. 2E). Results were unchanged in the multivariate analysis: PET/CT‐high (relative HR, 2.5; 95% CI, 1.2–5.2), HRRmt (relative HR, 2.1; 95% CI, 1.0–4.4) and TTs‐positive (relative HR, 2.2; 95% CI, 1.1–4.4) were significantly associated with poor outcome (Fig. 2F and Table 2).

**Table 2:** Cox regression analysis of PFS, *n* = 74. HR, hazard ratio; HRR, homologous recombination repair; PFS, progression‐free survival; TTs, therapeutic targets.

Characteristic	Multivariate analysis	
	HR (95% CI)	*P* value
Model 1		
Status of ‘PET/CT' (high vs. low)	2.5 (1.2–5.2)	**0.007**
Prognosis category (good vs. intermediate)^a^	1.1 (0.5–2.5)	0.785
History of docetaxel (yes vs. no)	2.8 (1.3–5.9)	**0.007**
Model 2		
Status of ‘HRR' (mt vs. wt)	2.1 (1.0–4.4)	**0.041**
Prognosis category (good vs. intermediate)	1.6 (0.7–3.5)	0.229
History of docetaxel (yes vs. no)	2.5 (1.2–5.3)	**0.017**
Model 3		
Status of ‘PTEN' (intact vs. loss)	1.2 (0.5–3.0)	0.689
Prognosis category (good vs. intermediate)	1.5 (0.7–3.3)	0.312
History of docetaxel (yes vs. no)	2.8 (1.3–5.9)	**0.009**
Model 4		
Status of ‘TTs' (positive vs. negative)	2.2 (1.1–4.4)	**0.020**
Prognosis category (good vs. intermediate)	1.3 (0.6–2.8)	0.553
History of docetaxel (yes vs. no)	2.8 (1.3–5.9)	**0.007**

*P* value of <0.05 were presented in bold.

a No patients were allocated to the poor prognosis category.

### 3.4 Exploratory analysis of prognostic value of TTs

**Fig. 3:**
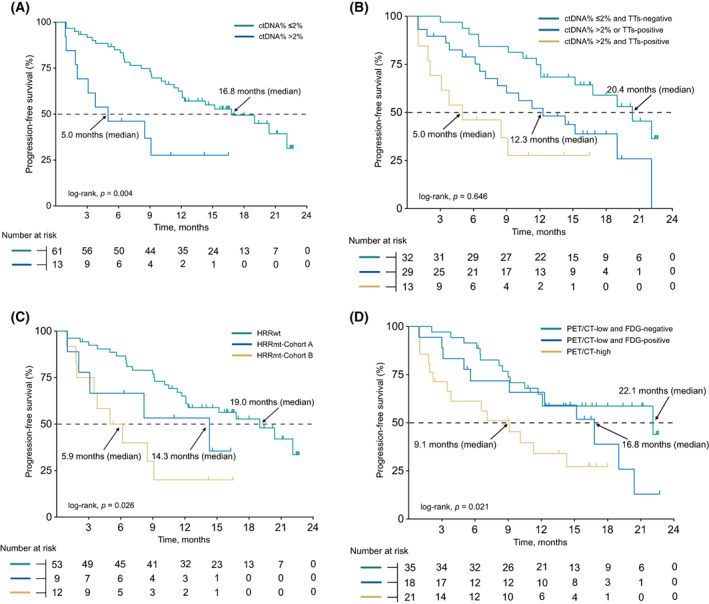
Exploratory analysis of prognostic value of TTs. (A) Progression‐free survival on abiraterone treatment in patients with ctDNA% > 2% or ≤ 2%. (B) Kaplan–Meier survival curves for patients stratified into three groups based on ctDNA% and status of TTs. (C) Kaplan–Meier survival curves for patients stratified into three groups based on status of HRR genes. (D) Kaplan–Meier survival curves for patients stratified into three groups based on status of ‘PET/CT' and PSMA‐/FDG lesion. *Patients with (without) PSMA‐/FDG lesion was defined as FDG‐positive (FDG‐negative) patients. ctDNA, circulating tumor DNA; HRR, homologous recombination repair; TTs, therapeutic targets.

A ctDNA% of > 2% was proved to be significantly associated with worse outcomes of abiraterone of enzalutamide [30]. Similarly, we found patients with ctDNA% > 2% (*n* = 13) had a shorter PFS than patients ctDNA% ≤ 2% (*n* = 61; median 5.0 vs. 16.8 months; HR, 2.8; 95% CI, 0.9–8.1; *P* = 0.004; Fig. 3A). The combination of ctDNA% with TTs provided significant survival stratification. After dividing patients into three subgroups according to the status of TTs and ctDNA%, we found that patients with ctDNA% ≤ 2% and negative TTs had a significantly longer PFS than the patients with ctDNA% > 2% or positive TTs (median 20.4 vs. 12.3 months; HR, 0.5; 95% CI, 0.2–0.9; *P* = 0.036; Fig. 3B).

Cohort B patients (*n* = 9) had a significantly shorter PFS than HRRwt group patients (median 5.6 vs. 19.0 months; HR, 3.5; 95% CI, 1.1–10.9, *P* < 0.001). However, no statistical difference was noted between the cohort A patients (*n* = 12) and the HRRwt group patients (median 14.3 vs. 19.0 months; HR, 1.9; 95% CI, 0.6–6.4, *P* = 0.178), which may be caused by the limited number of cohort A patients (Fig. 3C).

PET/CT low group was divided into two groups according to the status PSMA−/FDG+ lesion. We found PSMA−/FDG+ lesion improved stratification of progression risk (*P* = 0.021; Fig. 3D).

## 4 Discussion

In this study, we explored the stratification and prognosis of Chinese mCRPC patients according to three TTs. Positive TTs were observed in 58% of patients, of which 29% would have been candidates for poly ADP‐ribose polymerase inhibitors (PARPi) and 16% for an AKT‐inhibitor. Stratified by PSA level, 24% of mCRPC patients with PSA < 20 ng·mL^−1^ would have met the specific TT criteria of the TheraP trial, and the percentage was raised to 50% when PSA ≥ 20 ng·mL^−1^. Patients with positive TTs had poorer PFS for abiraterone. In addition, ctDNA% and PSMA−/FDG+ lesion improved stratification of progression risk.

Our study provides a comprehensive overview of promising TTs in an under‐representative population in precision oncology [31] (Table S4). The percentage of patients from East Asia involved in the TheraP, IPATential150, and PROfound trials is 0%, 19.07%, and 26.01%, respectively. In the on‐going trials of TTs‐driven trials, only trials of PARPi inhibitors (67%, 4/6) prepare to involve patients from East Asia. Selection bias and recall bias were minimized as compared to those in previous retrospective reports [32]. Unlike PROfound trial, the most frequently altered gene among the HRR genes in our cohort was *CDK12*. However, a recent multi‐institutional study, which identified the largest ctDNA genomic landscape of Chinese patients with mCRPC so far, also showed the most frequently altered gene among the HRR genes was *CDK12* (15.4%). [33] The rates of PET/CT‐high and HRRmt in Chinese mCRPC patients were comparable with western counterparts [4, 5], while the prevalence of PTEN‐loss was apparently lower in the East Asian population [6]. Furthermore, detailed analyses of the distribution of TTs provide intriguing insights for mCRPC management. First, we found a mutual independence among different TTs. Although previous studies have suggested that there is higher PSMA expression in patients with DNA repair defects [34] or activation of the PI3K‐Akt–mTOR pathway [35], our PET/CT imaging data did not support these hypotheses. Second, the correlation between clinicopathological features and TTs suggested several potential indicators of positive TTs, which may reduce the cost and time of TTs status screening. Finally, although the TheraP trial used PSA ≥ 20 ng·mL^−1^ as the inclusion criteria, we found that a remarkable proportion (24%) of mCRPC patients with PSA < 20 ng·mL^−1^ fulfilled the dual‐tracer PET/CT criteria. Of the 21 PET/CT‐high high group patients, patients with ≥ 20 ng·mL^−1^ had a significantly shorter PFS than patients with < 20 ng·mL^−1^ (median 2.2 vs. 11.3 months, *P* < 0.01; Fig. S1). This may be cause by the statistically positive correlation between the higher PSA and the greater likelihood of positive TTs.

With the success of the PROfound, TheraP, and IPATential150 trials, an unmet question is the outcome of patients who did not fulfill any of these inclusion criteria. Although we used the TTs criteria of these three trials merely and ignored other enrollment requirements, our observations had some significant implications. We found a better PFS of patients with TT‐negative disease when treated with abiraterone. PTEN loss by IHC [36, 37] and aberration in HRR genes [30] have been reported to be related to worse outcomes in the abiraterone‐treated patient, while there are controversial data regarding the prognostic value of the PSMA+FDG PET/CT in mCRPC. Thang et al. found a poor survival outcome for patient ineligible for Lu‐labeled PSMA radioligand therapy [38, 39]. Half of their patients had low PSMA expression and the other half had PSMA−/FDG+ disease. The poor prognosis of FDG only disease was in accordance with the data from Fox et al. [40]. Both studies evaluated patients with higher tumor volume than those in our reports (54.7%) which may impact the prognostic value of distinctive imaging phenotypes. In the current study, exploratory analyses showed that patients with low PSMA uptake had numerically better survival than those with PSMA−/FDG+ lesions. Therefore, it is interesting to longitudinal monitoring PSMA−/FDG+ disease under treatment of novel androgen receptor signaling inhibitors. Moreover, patients with low ctDNA% had significantly better outcomes as compared to those with high ctDNA%. This may be because of the enrichment of positive TTs in patients with ctDNA% > 2%.

While novel insights can be gathered from our analysis, several limitations exist. First, although we did not find different TTs distributions according to CRPC treatment lines, 57.5% of our patients were first‐line mCRPC which did not reproduce the enrolment scenario in the PROfound and TheraP trial [4, 5]. The therapeutic value of potential targets may be different among mCRPC treatment lines, indicating that our findings should be interpreted with caution. Second, unlike the PROfound trial, we used the ctDNA rather than tumor tissue to identify HRR genotyping. However, HRR gene status was found to be concordant between serial ctDNA‐positive sample collected in the mCRPC setting and archival primary tissue taken at cancer diagnosis [41, 42]. In addition, the ctDNA% in our cohort was low due to the low PSA level at enrolment. Therefore, it is possible that a subset of patients with somatic HRR alterations were not detected. Moreover, we did not include *AR* status into the abiraterone treatment efficacy analyses. Nevertheless, our study prompts the precision treatment approach for mCRPC patients according to the TTs, which provided the basis for an ongoing umbrella study (ChiCTR2100044833) with dynamic parallel treatment arms.

## 5 Conclusions

We reported the prevalence of TTs in a Chinese mCRPC cohort. Nearly 60% of patients, who had a poor prognosis on abiraterone, can be allocated to current precision treatment. Pending validation, the study provided a selective treatment approach including both precision and conventional treatment to the overall mCRPC patients and suggests the need for the early screen of TTs.

## Conflict of interest

The authors declare no conflict of interest.

## Author contributions

YZ, DY and SS conceptualized and designed the study. JP, JZ, XN, CL, HG and YW involved in acquisition of data. JP, JZ, XN, HG, BW, JW, TZ, QW analyzed and interpreted the data. JP, JZ, BW, CL, YZ, DY and SS wrote, reviewed and/or revised the manuscript. SJF, YZ and DY supervised the study.

### Peer review

The peer review history for this article is available at https://publons.com/publon/10.1002/1878‐0261.13320.

## Supporting information


**Fig. S1.** Kaplan–Meier survival curves according to the PSA level (*n* = 21).
**Table S1.** Inclusion rates after screen and proportion of patients from East Asia in three biomarker‐driven trials and FUSCC cohort.
**Table S2.** Association among three TTs in the cohort.
**Table S3.** Treatment decision of the rest patients, *n* = 32.
**Table S4.** On‐going clinical trials driven by TT for the treatment of mCRPC.

## Data Availability

The datasets analyzed during this study are available from the corresponding author on reasonable request.
