# Supplementary material for: The prevalence and prognosis of next‐generation therapeutic targets in metastatic castration‐resistant prostate cancer
Source: Mol Oncol. 2022 Oct 20;16(22):4011–22. doi: 10.1002/1878-0261.13320 (PMC9718110; doi:10.1002/1878-0261.13320)
Supplement: Supplementary file 1 — Fig. S1. Kaplan–Meier survival curves according to the PSA level (n = 21). Table S1. Inclusion rates after screen and proportion of patients from East Asia in three biomarker‐driven trials and FUSCC cohort. Table S2. Association among three TTs in the cohort. Table S3. Treatment decision of the rest patients, n = 32. Table S4. On‐going clinical trials driven by TT for the treatment of mCRPC. [file MOL2-16-4011-s001.docx]

**Supporting Information for:**

**The prevalence and prognosis of next-generation** **therapeutic targets in metastatic castration-resistant prostate cancer**

Jian Pan^1,2,3,#^, Jinou Zhao^1,2,3,#^, Xudong Ni^1,2,3^, Hualei Gan^2,3^, Yu Wei^1,2,3^, Junlong Wu^1,2,3^, Tingwei Zhang^1,2,3^, Qifeng Wang^2,3^, Stephen J. Freedland^5,6^, Beihe Wang^1,2,3,*^, Shaoli Song^3,4,*^, Dingwei Ye^1,2,3,*^, Chang Liu^3,4,*^, Yao Zhu^1,2,3,*^

# These authors contributed equally to this work

* Corresponding authors

1. Department of Urology, Fudan University Shanghai Cancer Center, Shanghai, China
2. Shanghai Genitourinary Cancer Institute, Shanghai, China
3. Department of Oncology, Shanghai Medical College, Fudan University, Shanghai, China
4. Department of Pathology, Fudan University Shanghai Cancer Center, Shanghai China
5. Department of Nuclear Medicine, Fudan University Shanghai Cancer Center, Shanghai China
6. Department of Surgery, Division of Urology and Samuel Oschin Comprehensive Cancer Institute, Cedars-Sinai Medical Center, Los Angeles, CA
7. Urology Section, Department of Surgery, Veterans Affairs Medical Center, Durham, NC

**Correspondence**

Yao Zhu, Department of Urology, Fudan University Shanghai Cancer Center, Shanghai 200032, People’s Republic of China

Tel: 86-21-64175590-2800

Email: zhuyao@fudan.edu.cn

and

Chang Liu, Department of Nuclear Medicine, Fudan University Shanghai Cancer Center, Shanghai 200032, People’s Republic of China

Tel: 86-21-34205373

Email: lcggtt@163.com

and

Dingwei Ye, Department of Urology, Fudan University Shanghai Cancer Center, Shanghai 200032, People’s Republic of China

Tel: 86-21-64175590-2805

Email: dwye.shca@gmail.com

and

Shaoli Song, Department of Nuclear Medicine, Fudan University Shanghai Cancer Center, Shanghai 200032, People’s Republic of China

Tel: 86-21-34205373

Email: shaoli-song@163.com

and

Beihe Wang, Department of Urology, Fudan University Shanghai Cancer Center, Shanghai 200032, People’s Republic of China

Tel: 86-21-64175590-2800

Email: med_wangbh@163.com

**Table of Contents**

**1. Supplementary figures**

Fig. S1. Supplementary Figure 1. Kaplan-Meier survival curves according to the PSA level (*n* = 21).

**2. Supplementary tables**

Table S1. Inclusion rates after screen and proportion of patients from East Asia in three biomarker-driven trials and FUSCC cohort.

Table S2. Association among three TTs in the cohort.

Table S3. Treatment decision of the rest patients, *n* = 32.

Table S4. On-going clinical trials driven by TT for the treatment of mCRPC.

**Figure S1**. Kaplan-Meier survival curves according to the PSA level (*n* = 21).


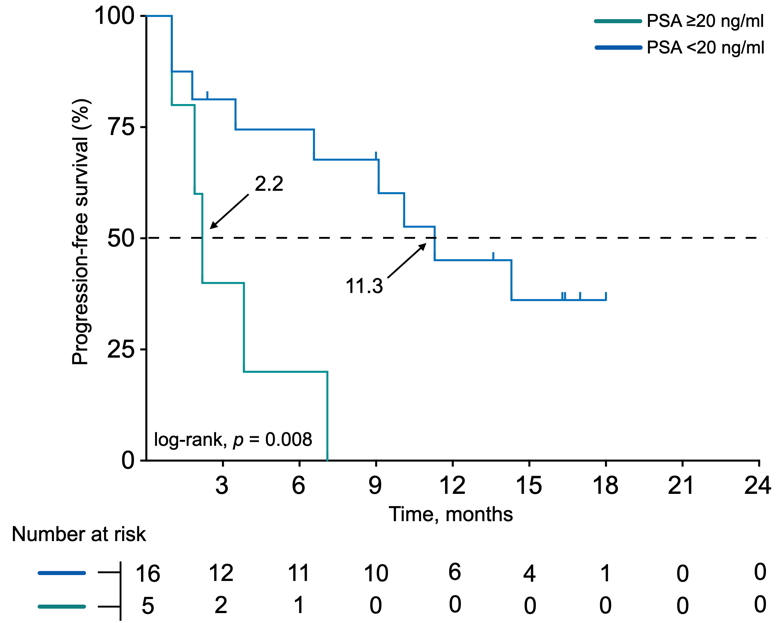


PSA = prostate-specific antigen.

**Table S1**. Inclusion rates after screen and proportion of patients from East Asia in three TTs-driven trials and FUSCC cohort.

| Clinical trial | TTs definition | | Inclusion or Stratification  Rates after Screen | % of patients from East Asia |
| --- | --- | --- | --- | --- |
| TheraP trial | high PSMA disease and no FDG-discordant disease | PSA≥20 | 72.51% (211/291) | 0 |
| FUSCC cohort |  | PSA≥20 | 50.00% (12/24) | 100% |
|  |  | PSA<20 | 24.39% (20/82) | 100% |
| IPATential150 trial | with PTEN-loss tumors | | 40.15% (208/518) | 19.07% (210/1101) |
| FUSCC cohort |  |  | 16.04% (17/106) | 100% |
| PROfound trial | ≥1 germline and/or somatic alterations in 15 HRR genes | | 27.87% (778/2792) | 26.01% (101/387) |
| FUSCC cohort |  |  | 29.25% (31/106) | 100% |

TTs: therapeutic targets; FUSCC: Fudan University Shanghai Cancer Center; HRR: homologous recombination repair

**Table S2**. Association among three TTs in our cohort

| TTs | Overall | PET/CT | | *p* | HRR | | *p* | PTEN | | *p* |
| --- | --- | --- | --- | --- | --- | --- | --- | --- | --- | --- |
|  | (*n* = 106) |  |  |  |  |  |  |  |  |  |
|  |  | high | low |  | mt | wt |  | loss | intact |  |
|  |  | 32 | 74 |  | 31 | 75 |  | 17 | 89 |  |
| PET/CT |  |  |  |  |  |  |  |  |  |  |
| high | 32 |  |  |  | 10 | 22 | 0.818 | 6 | 26 | 0.774 |
| low | 74 |  |  |  | 21 | 53 |  | 11 | 63 |  |
| HRR |  |  |  |  |  |  |  |  |  |  |
| mt | 31 | 10 | 21 | 0.818 |  |  |  | 3 | 28 | 0.384 |
| wt | 75 | 22 | 53 |  |  |  |  | 14 | 61 |  |
| PTEN |  |  |  |  |  |  |  |  |  |  |
| loss | 17 | 6 | 11 | 0.774 | 3 | 14 | 0.384 |  |  |  |
| intact | 89 | 26 | 63 |  | 28 | 61 |  |  |  |  |

TTs: therapeutic targets; HRR: homologous recombination repair; IHC: immunohistochemistry.

**Table S3**. Treatment decision of the rest patients (*n* = 32).

| ID | Previous treatment | PET/CT status | PTEN status | HRR status | TTs status | Following treatment |
| --- | --- | --- | --- | --- | --- | --- |
| 1 | ADT | Low | Intact | Wild | Negative | Radiotherapy |
| 2 | ADT | Low | Intact | Wild | Negative | Radiotherapy |
| 3 | ADT | Low | **Loss** | Wild | **Positive** | Radiotherapy |
| 4 | ADT + abiraterone | Low | Intact | Wild | Negative | Docetaxel |
| 5 | ADT + abiraterone | Low | Intact | Wild | Negative | Docetaxel |
| 6 | ADT + abiraterone | Low | Intact | Wild | Negative | Docetaxel |
| 7 | ADT + abiraterone | Low | Intact | Wild | Negative | Docetaxel |
| 8 | ADT + abiraterone | Low | Intact | Wild | Negative | Docetaxel |
| 9 | ADT + abiraterone | Low | Intact | **Mutation** | **Positive** | Docetaxel |
| 10 | ADT + abiraterone | **High** | Intact | Wild | **Positive** | Docetaxel |
| 11 | ADT + abiraterone | **High** | **Loss** | Wild | **Positive** | Docetaxel |
| 12 | ADT + abiraterone | **High** | Intact | Wild | **Positive** | Docetaxel |
| 13 | ADT + abiraterone | **High** | Intact | **Mutation** | **Positive** | Docetaxel |
| 14 | ADT + abiraterone | Low | Intact | **Mutation** | **Positive** | Docetaxel |
| 15 | ADT + abiraterone | **High** | Intact | **Mutation** | **Positive** | Olaparib |
| 16 | ADT + abiraterone | Low | Intact | Wild | Negative | Olaparib |
| 17 | ADT + abiraterone | **High** | Intact | Wild | **Positive** | Olaparib |
| 18 | ADT + abiraterone + docetaxel | Low | Intact | Wild | Negative | Docetaxel + carboplatin |
| 19 | ADT + abiraterone + docetaxel | **High** | **Loss** | **Mutation** | **Positive** | Docetaxel + carboplatin |
| 20 | ADT + abiraterone + docetaxel | Low | Intact | Wild | Negative | Docetaxel + carboplatin |
| 21 | ADT + abiraterone + docetaxel | **Low** | Intact | Wild | **Negative** | Docetaxel + carboplatin |
| 22 | ADT + abiraterone + docetaxel | Low | **Loss** | Wild | **Positive** | Olaparib |
| 23 | ADT + abiraterone + docetaxel | **High** | Intact | **Mutation** | **Positive** | Olaparib |
| 24 | ADT + abiraterone + docetaxel | **High** | Intact | Wild | **Positive** | Olaparib |
| 25 | ADT + abiraterone + docetaxel | Low | Intact | **Mutation** | **Positive** | Olaparib |
| 26 | ADT + abiraterone + docetaxel | Low | Intact | **Mutation** | **Positive** | Olaparib |
| 27 | ADT + abiraterone + docetaxel | Low | **Loss** | Wild | **Positive** | Olaparib |
| 28 | ADT + abiraterone + docetaxel | Low | Intact | **Mutation** | **Positive** | Olaparib |
| 29 | ADT + abiraterone + docetaxel | **High** | Intact | Wild | **Positive** | Olaparib |
| 30 | ADT + abiraterone + docetaxel | **High** | Intact | Wild | **Positive** | ^177^Lu-PSMA-617 |
| 31 | ADT + abiraterone + docetaxel | Low | **Loss** | Wild | **Positive** | Pembrolizumab |
| 32 | ADT + docetaxel | Low | Intact | **Mutation** | **Positive** | Olaparib + abiraterone |

TTs: therapeutic targets; HRR: homologous recombination repair; ADT: androgen deprivation therapy.

**Table S4**. On-going trials driven by TTs for the treatment of mCRPC

| Clinical trial^a^ | Study type | Treatment arms | Involves East Asian patients or not |
| --- | --- | --- | --- |
| On-going trials of **PARP inhibitors** for the treatment of mCRPC | | | |
| PROpel  (NCT03732820) | Phase 3,  randomised, double blind | Olaparib plus abiraterone versus placebo plus abiraterone  (estimated *n* = 904) | Yes |
| TALAPRO-2  (NCT03395197) | Phase 3,  randomised, double blind | Talazoparib plus enzalutamide versus placebo plus enzalutamide(estimated *n* = 1037） | Yes |
| CASPAR  (NCT04455750) | Phase 3,  randomised, double blind | Rucaparib plus enzalutamide versus placebo plus enzalutamide  (estimated *n* = 1002） | No |
| MAGNITUDE (NCT03748641) | Phase 3,  randomised, double blind | Niraparib plus abiraterone and prednisone versus placebo plus abiraterone and prednisone  (estimated *n* = 1000) | Yes |
| TRITON3  (NCT02975934) | Phase 3,  open label, double blind | Rucaparib versus physicians’ choice of enzalutamide or abiraterone or docetaxel  (estimated *n* = 400) | No |
| KEYLYNK-010 (NCT03834519) | Phase 3,  randomised, open label | Olaparib plus pembrolizumab versus abiraterone plus prednisone or enzalutamide  (estimated *n* = 780) | Yes |
| On-going trials of **radiolabelled small molecules that bind to PSMA** for the treatment of mCRPC | | | |
| NCT03724747 | Phase 1  non-randomised, open label | BAY 2315497 Injection  (estimated *n* = 157) | No |
| NCT03490838 | Phase 1/2,  non-randomised, open label | 177Lu-PSMA-R2(a thorium-227 labeled immuno-conjugate, PSMA)  (estimated *n* = 96） | No |
| ENZA-p  (NCT04419402) | Phase 2,  randomised, open label | Lu PSMA-617 plus enzalutamide versus enzalutamide  (estimated *n* = 160) | No |
| NCT04663997 | Phase 2,  randomised, open label | Lu PSMA-617 versus docetaxel  (estimated *n* = 200） | No |
| NCT03939689 | Phase 2,  randomised, open label | I-131-1095 plus enzalutamide versus enzalutamide  (estimated *n* = 175) | No |
| SPLASH  (NCT04647526) | Phase 3,  randomised, open label | [Lu-177]-PNT2002 versus abiraterone/prednisone or enzalutamide  (estimated *n* =415) | No |
| PSMAfore  (NCT04689828) | Phase 3,  randomised, open label | 177Lu-PSMA-617 versus physicians’ choice of ARDT  (estimated *n* =495) | Not known |
| VISION  (NCT03511664) | Phase 3,  randomised, open label | 177Lu-PSMA-617 plus BS/BSOC versus BS/BSOC  (estimated *n* =831) | No |
| On-going trials of **AKTi** for the treatment of mCRPC | | | |
| Ice-CAP  (NCT03673787) | Phase 1,  non-randomised, open label | Ipatasertib plus atezolizumab  (estimated *n* = 51) | No |
| AZD5363  (NCT04087174) | Phase 1,  non-randomised, open label | Capivasertib plus enzalutamide/abiraterone  (estimated *n* = 27） | No |
| NCT04404140 | Phase 1b,  open label | Ipatasertib/atezolizumab plus docetaxel  (estimated *n* = 50） | No |
| NCT02833883 | Phase 1b,  open label | Enzalutamide Plus CC-115  (estimated *n* = 40) | No |
| NCT04060394 | Phase 2,  randomised | LAE001/prednisone plus afuresertib and docetaxel/prednisone plus afuresertib  (estimated *n* = 74) | No |

^a^ A systematic ClinicalTrials.gov search was conducted in May 2021.

TTs: therapeutic targets; mCRPC: metastatic castration-resistant prostate cancer; PARP: poly ADP-ribose polymerase; PSMA: prostate- specific membrane antigen; BO/BSOC = Best supportive/best standard of care; AKTi: Akt-inhibitors.
